# Wicked problems and a ‘wicked’ solution

**DOI:** 10.1186/s12992-018-0353-x

**Published:** 2018-04-13

**Authors:** Helen L. Walls

**Affiliations:** Faculty of Public Health and Policy, London School of Hygiene and Tropical Medicine, 15-17 Tavistock Place, London, WC1H 9SH UK

**Keywords:** Governance, Policy, Wicked problems

## Abstract

**Background:**

‘Wicked’ is the term used to describe some of the most challenging and complex issues of our time, many of which threaten human health. Climate change, biodiversity loss, persisting poverty, the advancing obesity epidemic, and food insecurity are all examples of such wicked problems. However there is a strong body of evidence describing the solutions for addressing many of these problems. Given that much is known about how many of these problems could be addressed – and given the risks of not acting – what will it take to create the ‘tipping point’ needed for effective action?

**Main body:**

A recent (2015) court ruling in The Hague held that the Dutch government’s stance on climate change was illegal, ordering them to cut greenhouse gas emissions by at least 25% within 5 years (by 2020), relative to 1990 levels. The case was filed on behalf of 886 Dutch citizens, suing the government for violating human rights and climate changes treaties by failing to take adequate action to prevent the harmful impacts of climate change. This judicial ruling has the potential to provide a way forward, inspiring other civil movements and creating a template from which to address other wicked problems.

**Conclusion:**

This judicial strategy to address the need to lower greenhouse gas emissions in the Netherlands is not a magic bullet, and requires a particular legal and institutional setting. However it has the potential to be a game-changer – providing an example of a strategy for achieving domestic regulatory change that is likely to be replicable in some countries elsewhere, and providing an example of a particularly ‘wicked’ (in the positive, street-slang sense of the word) strategy to address seemingly intractable and wicked problems.

## Background

Policymakers have had great difficulty, almost by definition, responding effectively to complex or what have been termed ‘wicked’ problems, many of which threaten human health. These challenges are a result of the defining features of wicked problems – their complex, numerous and sometimes undefined causes which are often globalized, their contested understandings and framings among stakeholders with different and strongly held beliefs and values, and their need for collective action sometimes on a global level but lack of simple planning responses [1, 2]. Climate change, biodiversity loss, persisting poverty, the growing obesity epidemic, and food insecurity are all examples of such wicked problems.

With obesity, for example, no country to date has reversed its obesity epidemic [3], despite a considerable amount of attention devoted to the importance of this issue [4], and a growing evidence base of the effectiveness of various solutions [5]. To a significant extent, this is due to the ideological debates surrounding the issue, often driven by industry actors and their well-funded lobbies defending vested economic interests and deliberately exploiting the complex or wicked nature of the problem, and thereby creating further uncertainty about causes and consequences. These debates include those of personal versus collective responsibility for action, supply- versus demand-type explanations for consumption of unhealthy food, the relative roles of physical inactivity and diet in weight gain, government regulation versus industry self-regulation, and treatment versus prevention priorities [3]. Similar debates and controversies surround discussions of the nature of, and how best to respond to, other wicked problems, and there is a documented industry strategy to influence country positions, create debate and confusion, and delay government regulatory responses [6, 7]. Given that many of these problems have a strong body of evidence in support of various intervention solutions for addressing them – and given the risks of not acting – what will it take to create the ‘tipping point’ needed for effective action?

## Main text

We may not have to wait for the respective apocalypses. A recent (2015) court ruling in The Hague, using the principles of tort law to address civil wrong-doings, held that the Dutch government’s stance on climate change was illegal, and ordered them to cut greenhouse gas emissions by at least 25% by 2020, relative to 1990 levels. Given the threat posed by climate change, the ruling included that cutting emission by a lesser among of 14% to 17% by 2020, as were the government plans at the time, was unlawful. Urgenda, an environmental non-profit organisation, filed the case on behalf of 886 Dutch citizens, suing the government for violating human rights by failing to take adequate action to prevent the harmful impacts of climate change [8, 9]. Thus, after two decades of international negotiations on climate change, heated national policy debates in many countries, and little policy change to address greenhouse gas emissions – and certainly not on the scale required – this judicial ruling may provide a way forward for addressing this and other wicked problems.

The ruling uses a combination of Dutch civil law (the principle of a government’s duty of care) and existing human rights and climate changes treaties – a legal basis with potential to be used in courts elsewhere. Similar cases are in recent years being prepared in Belgium, Norway and the Philippines. *The Guardian* speculated as to whether the Dutch judgement could inspire a global civil movement to address climate change [8]. However the case may also inspire civil movements and create a template from which to address other wicked problems.

Public health experts have often suggested that addressing wicked problems affecting health will ultimately require government regulation and that, given governmental reluctance to act, and short of catastrophe, the impetus for such regulation will come from civil society [10]. The Dutch court ruling may be the ‘tipping point’ needed to force governmental regulatory change – something often hampered by conflicting stakeholder views and strong industry lobbies – by raising the political priority of the issue, and cutting through gridlock caused by institutional power imbalances by handing government a strong and legally binding mandate on which to act. Legal rulings ordering governments to address issues such as biodiversity loss, persisting policy, food insecurity or obesity may sound far-fetched, but are perhaps no less radical than how other important advances may have once been perceived, for example the introduction of sanitary law in nineteenth-century Britain [10]. But there are limitations. This legal strategy requires a well-functioning, democratic government, an effective judicial system, and a mobilised civil society. Not all countries have the legal system and requisite institutions within which this type of case could be brought against a government. It is also unclear exactly how the court will enforce its ruling. However the ruling does provide an example of a strategy for achieving domestic regulatory change that is likely to be replicable in some countries elsewhere.

## Conclusion

This judicial strategy to address the need to lower greenhouse gas emissions in one country is not a magic bullet, but it has the potential to be a game-changer – providing an example of a strategy for achieving domestic regulatory change that is likely to be replicable in some countries elsewhere, and providing an example of a particularly ‘wicked’ (in the positive, street-slang sense of the word) strategy to address seemingly intractable and wicked problems.
